# Association of *IL-6* -174G>C (rs1800795) polymorphism with cervical cancer susceptibility

**DOI:** 10.1042/BSR20181071

**Published:** 2018-09-14

**Authors:** Hai-Xia Duan, You-Yi Chen, Juan-Zi Shi, Nan-Nan Ren, Xiao-Juan Li

**Affiliations:** 1 Department of Reproduction Gynecology, Northwest Women and Children’s Hospital, Shannxi 710003, Xi’an, China; 2 Department of Reproductive Medical center, Xi’an No. 4 Hospital, Shannxi 710004, Xi’an, China; 3 Reproductive Center, Northwest Women and Children’s Hospital, Shaanxi 710003, Xi’an, China; 4 Department of Inspection, Xi’an No. 4 Hospital, Shaanxi 710004, Xi’an, China; 5 Department of Reproduction Gynecology, Northwest Women and Children’s Hospital, Shaanxi 710003, Xi’an, China

**Keywords:** IL-6, -174G>C, cervical cancer, meta-analysis, polymorphism, risk

## Abstract

Interleukin-6 (IL-6) is a multifunctional cytokine that has been implicated in the etiology of cancer. Several case–control studies have been conducted to assess the association of *IL-6* -174G>C (rs1800795) polymorphism with the risk of cervical cancer, yet with conflicting conclusions. To derive a more precise estimation of the relationship, we performed this meta-analysis updated to June 2018. A total of seven original publications were identified covering *IL-6* -174G>C (rs1800795) polymorphism. Odds ratios (ORs) and 95% confidence intervals (CIs) were used to assess the relationship strengths. Statistically significant relationship was observed between *IL-6* -174G>C polymorphism and cervical cancer risk (OR = 0.61, 95% CI: 0.40–0.94 for GG vs. CC, and OR = 0.77, 95% CI: 0.64–0.93 for G vs. C). Moreover, the significant association was found among Asians (OR = 0.46, 95% CI: 0.29–0.75 for GG vs. CC, and OR = 0.70, 95% CI: 0.57–0.89 for G vs. C); hospital-based subgroup (OR = 0.53, 95% CI: 0.38–0.72 for GG vs. CC, and OR = 0.73, 95% CI: 0.61–0.87 for G vs. C); and Hardy–Weinberg equilibrium ≤0.05 (OR = 0.56, 95% CI: 0.37–0.86 for GG vs. GC, and OR = 0.66, 95% CI: 0.47–0.93 for G vs. C). This meta-analysis showed the evidence that the *IL-6* -174G>C polymorphism was a low-penetrance susceptibility variant for cervical cancer. Further large-scale case–control studies are needed to confirm these results.

## Introduction

Cervical cancer ranks the second most malignant diseases in women. Cervical cancer accounts for 528,000 new cases and 266,000 deaths worldwide each year, according to GLOBOCAN 2012 [1]. Although early cervical cancer can be treated with radiation or surgery, metastatic cervical cancer is still incurable [2]. The etiology of cervical cancer has been largely explored, yet not totally elucidated. Growing evidence demonstrates that genetic variant also plays a critical role in the initiation and development of cervical cancer [3–5].

Previous studies have reported that inflammation plays a crucial role in the development of some cancers [6,7]. Inflammation favors tumorigenesis by damaging DNA [8], stimulating cell proliferation [9], and stimulating angiogenesis [10]. Interleukin-6 (IL-6) is a phosphorylated glycoprotein containing 185 amino acids. IL-6 is one of the most widely recognized cytokines associated with inflammation [11,12]. It regulates several important cellular pathways including: cell proliferation, differentiation, immune responses, invasion, metastasis as well as carcinogenesis [13–16].

The human *IL-6* gene, consists of five exons and four introns, is located on chromosome 7p21. Several SNPs of *IL-6* gene have been identified to be associated with cancer risk, but the most popular studied SNP is *IL-6* -174G>C (rs1800795) polymorphism [17–19]. The common single nucleotide polymorphism at position -174 (*IL-6* -174G>C, rs1800795) of the IL-6 gene promoter is thought to influence the binding of the glucocorticoid receptor and thus repress transcriptional activation [20,21]. Recently, many studies investigated the role of this polymorphism in the etiology of cervical cancer. However, the results of these studies remain conflicting. With the aim to measure the correlation, we performed this comprehensive meta-analysis by adopting all eligible studies.

## Materials and methods

### Publication search

The database web of science, Google Scholar, PubMed, EMBASE, CNKI, and Wanfang were used to search publications. The following keywords were adopted: “single nucleotide polymorphism or polymorphism or SNP or variant” and “*IL6* or *IL-6* or interleukin-6”, and “cervical cancer or cervical tumor or cervical neoplasm or cervical carcinoma”. Eligible studies were also extracted from the references from the obtained publications. The latest or the largest study was included in the final meta-analysis, if there exist two or more articles containing overlapping data [18,22]. The literature search was updated to June 2018.

### Eligibility criteria

Studies finally selected for analysis should meet all the following items: (1) unrelated case–control studies; (2) original epidemiological studies; (3) evaluation of the *IL-6* -174G>C polymorphism and cervical cancer risk; (4) information containing available genotype frequency that can help infer the odds ratios (ORs) and 95% confidence intervals (CIs). Exclusion criteria were as follows: (1) case only studies or case reports; (2) meta-analyses or reviews; (3) studies without detailed genotyping data; (4) duplicate publications.

### Data extraction

We arranged two authors (Hai-Xia Duan and You-Yi Chen) to extract data independently. The data include: first author’s surname, country, publication year, ethnicity, genotyping methods, the source of controls, and numbers of cases and controls with GG, GC and CC genotypes. The conflicting data would be confirmed by the third author.

### Statistical methods

STATA 11.0 software was adopted to perform all statistical analysis (Stata Corporation, College Station, TX). Goodness-of-fit χ^2^ test was used to evaluate deviation from Hardy–Weinberg equilibrium (HWE) for the genotypes of control subjects. We adopted three genetic models, homozygous model (GG vs. CC), heterozygous model (GG vs. GC), and allele comparison (G vs. C) to investigate the association between *IL-6* -174G>C polymorphism and cervical cancer risk. OR and its corresponding CI was used to determine the relationship between *IL-6* -174G>C and cervical cancer risk. Stratification analyses were also performed by ethnicity, source of control, and HWE in controls. Heterogeneity assumption was checked by a chi-square based Q-test. A *P*-value of more than 0.05 for the Q-test indicated a lack of heterogeneity among the studies, so the summary OR estimate of each study was calculated by the fixed-effects model (the Mantel–Haenszel method). Otherwise, the random effects model (DerSimonian and Laird method) was used. Sensitivity analysis was conducted to determine the stability of the results by sequentially excluding one study at a time and recalculating the pooled ORs and their corresponding 95% CIs. Furthermore, both the Begg’s funnel plot and the Egger’s linear regression test were used to assess the potential publication bias. All the statistics were two-sided with *P* value of <0.05 as significant findings.

## Results

### Study characteristics

**Figure 1:**
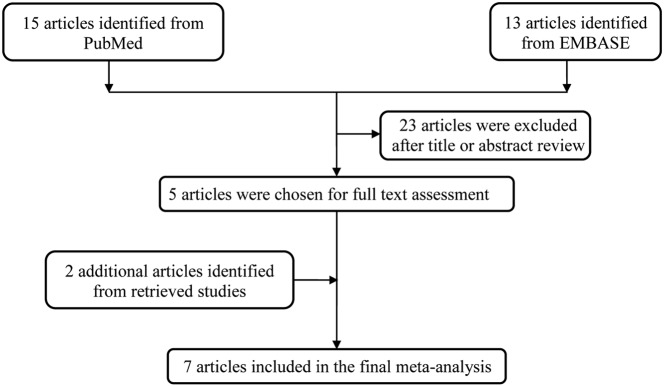
Flow diagram of the study selection process

Through literature search and selection based on the inclusion criteria, five articles were retrieved. Moreover, we also extracted two articles from the references of the retrieval articles. A flow chart was carefully identified of the search process in Figure 1. Finally, seven publications with 1452 cases and 2186 controls were used in the pooled analysis (Table 1) [23–29]. Among them, three studies focused on Asians and four on Caucasians. Five studies were hospital-based design and two were population-based design. The genotype frequencies of *IL-6* promoter in controls of five studies met the HWE expectation (*P*>0.05), except for two studies.

**Table 1:** The baseline characteristics of all qualified studies in this meta-analysis

Surname	Year	Country	Ethnicity	Control Source	Genotype method	Case				Control				HWE
						GG	GC	CC	All	GG	GC	CC	All	
Nogueira de Souza [23]	2006	Brazil	Caucasian	PB	PCR	24	32	0	56	148	102	3	253	0.001
Gangwar [28]	2009	India	Asian	HB	PCR	107	36	17	160	142	51	7	200	0.372
Grimm [27]	2011	Austria	Caucasian	HB	PCR	55	51	25	131	85	96	27	208	0.990
Lima Junior [29]	2012	Brazil	Caucasian	PB	PCR sequencing	72	39	4	115	67	37	11	115	0.093
Shi [24]	2014	China	Asian	HB	PCR-RFLP	160	253	105	518	181	259	78	518	0.349
Pu [26]	2016	China	Asian	HB	PCR	185	141	34	360	476	220	32	728	0.310
Sabrina Zidi [25]	2017	Tunisia	Caucasian	HB	PCR	81	25	6	112	133	25	6	164	0.002

Abbreviations: HB, hospital based; HWE, Hardy–Weinberg equilibrium; PB, population based; PCR, polymerase chain reaction; PCR-RFLP, PCR-restriction fragment length polymorphism.

### Quantitative synthesis

**Figure 2:**
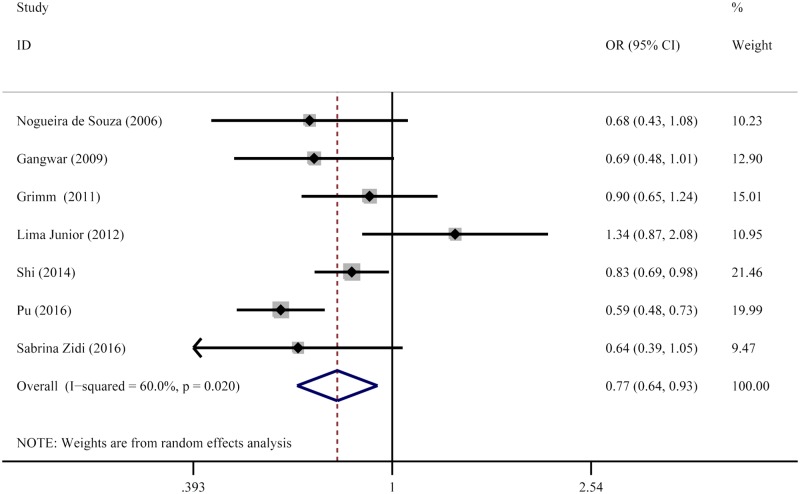
Forest plot of cervical cancer risk associated with the *IL-6* -174G>C (G vs. C) The squares and horizontal lines correspond to the study-specific OR and 95% CI. The area of the squares reflects the weight (inverse of the variance). The diamond represents the summary OR and 95% CI.

The summary results of meta-analysis were shown in Table 2 and Figure 2. Statistically significant relationship was observed between *IL-6* -174G>C polymorphism and cervical cancer risk in the two genetic models (OR = 0.61, 95% CI: 0.40–0.94 for GG vs. CC, OR = 0.77, and 95% CI: 0.64–0.93 for G vs. C). However, the -174G>C polymorphism was not associated with cervical cancer risk in heterozygous genetic model (OR = 0.81, 95% CI: 0.64–1.03 for GG vs. GC). In the stratified analysis by race, we found that a significantly relationship among Asians (OR = 0.46, 95% CI: 0.29–0.75 for GG vs. CC, and OR = 0.70, 95% CI: 0.57–0.89 for G vs. C). Moreover, we also detected other significant relationship among hospital-based subgroup (OR = 0.53, 95% CI: 0.38–0.72 for GG vs. CC, and OR = 0.73, 95% CI: 0.61–0.87 for G vs. C); and HWE ≤ 0.05 (OR = 0.56, 95% CI: 0.37–0.86 for GG vs. GC, and OR = 0.66, 95% CI: 0.47–0.93 for G vs. C).

**Table 2:** Meta-analysis of the association between IL-6 rs1800795 polymorphism and cervical cancer risk

Variables	No. of studies	Homozygous		Heterozygous		Allele	
		GG vs. CC		GG vs. GC		G vs. C	
		OR (95% CI)	*P*^het^	OR (95% CI)	*P*^het^	OR (95% CI)	*P*^het^
All	7	**0.61 (0.40–0.94)**	0.042	0.81 (0.64**–**1.03)	0.049	**0.77 (0.64–0.93)**	<0.001
Ethnicity							
Asian	3	**0.46 (0.29–0.75)**	0.099	0.81 (0.58**–**1.12)	0.050	**0.70 (0.57–0.89)**	0.053
Caucasian	4	0.99 (0.47**–**2.09)	0.179	0.81 (0.54**–**1.22)	0.092	0.86 (0.64**–**1.17)	0.093
Control source							
HB	5	**0.53 (0.38–0.72)**	<0.001	0.84 (0.64**–**1.10)	0.044	**0.73 (0.61–0.87)**	0.097
PB	2	2.60 (0.86**–**7.86)	0.091	0.73 (0.38-1.42)	0.100	0.96 (0.49**–**1.87)	0.035
HWE							
>0.05	5	0.61 (0.37**–**1.02)	0.012	0.89 (0.68**–**1.17)	0.050	0.81 (0.64**–**1.02)	0.006
≤0.05	2	0.66 (0.22**–**1.96)	0.696	**0.56 (0.37–0.86)**	0.706	**0.66 (0.47–0.93)**	0.875

Abbreviations: HB, hospital based; Het, heterogeneity; PB, population based.

Values in bold were significant findings if 95% CIs excluded 1.

### Heterogeneity and sensitivity analysis

**Figure 3:**
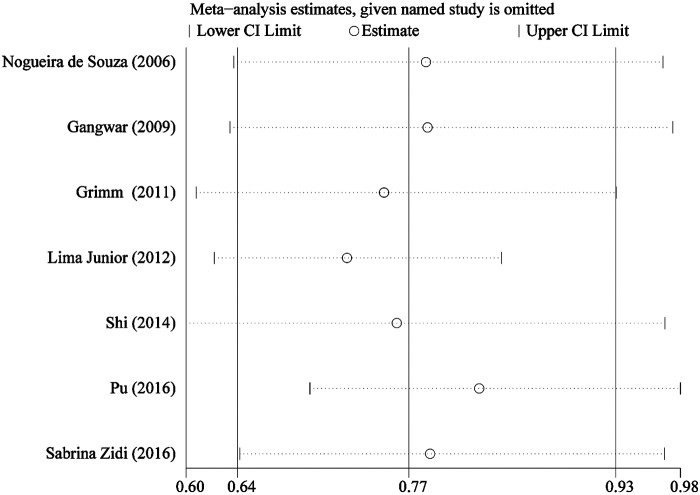
Sensitivity analysis of the summary OR coefficients on the association between *IL-6* -174G>C polymorphism and cervical cancer risk under allele comparison model

*Q* test and *I^2^* statistics were used to detect between-study heterogeneity. We detected significant heterogeneity among all three genetic models (*P*<0.001) in the pooled analysis. Hence, the random-effect model was used to generate wider CIs. In addition, sensitivity analysis was conducted to assess the stability of the results. However, no individual study affected the overall OR, since omission of any single study made no materially difference (Figure 3).

### Publication bias

**Figure 4:**
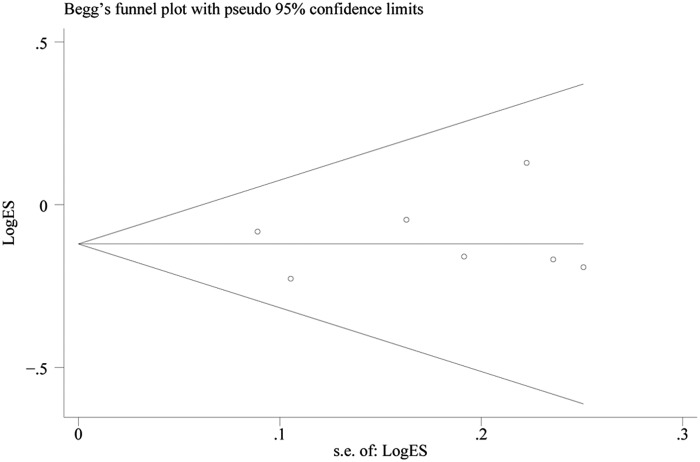
Begg’s funnel plot for publication bias test under allele comparison model Each point represents a separate study for the indicated association.

Each study in this meta-analysis was performed to evaluate the publication bias by both Begg’s funnel plot and Egger’s test. The shape of the funnel plots did not reveal any evidence of obvious asymmetry (Figure 4). In addition, statistical evidence of Egger’s test also provided the none-existence of publication bias among the studies (data not shown).

## Discussion

This meta-analysis explored the association between a commonly studied *IL-6* -174G>C polymorphism and cervical cancer risk. The obtained results suggested *IL-6* -174G>C polymorphism may influence cervical cancer risk in a low impact effect manner. To date, this meta-analysis represents the most powerful investigation in elucidating the role of *IL-6* -174G>C in cervical cancer risk.

Several studies have investigated the role of *IL-6* -174G>C polymorphism in cervical cancer risk. In 2006, Nogueira de Souza et al. [23] performed the first case–control study to assess whether *IL-6* polymorphisms would impact the risk of developing cervical cancer. Their study included 56 patients with cervical carcinoma and 253 population-based control subjects from Brazilian women. Their results suggested that women carrying at least one C genotype in their *IL-6* promoter region (-174G>C) are at higher risk of developing cervical cancer. In 2009, Gangwar et al. [28] also found a significant association of the *IL-6* -174 CC genotype with risk of cervical cancer (OR = 3.16; *P*=0.014) in Indian population. However, Grimm et al. failed to detect relationship between *IL-6* -174G>C polymorphism and the risk for cervical cancer [27]. A recent study by Pu et al. [26] found that cervical cancer risks were significantly higher in carriers of C allele of rs1800795 polymorphism than those with GG genotype. By now, only one meta-analysis by Liu et al. [30] was conducted to explore the association between *IL-6* -174G>C and cervical cancer risk. This meta-analysis was updated to July 2015 with five publications included (1210 cases and 1525 controls). Their results showed that the C genotype of interleukin 6 rs1800795 is associated with higher cervical cancer risk. However, due to the small sample size of the included studies, they did not perform subgroup analyses to clarify its association with *IL-6* gene.

To provide a robust clarification, we performed the updated meta-analysis by involving all the eligible studies published. Overall, our analysis indicates that the genotypes of *IL-6* -174G>C polymorphism are associated with cervical cancer risk. Such phenomenon might be due to that rs1800795 of the *IL-6* gene promoter influences the binding of the glucocorticoid receptor and thus represses transcriptional activation, which lead to the development of cervical cancer. Subgroup analysis by race suggested that C genotype significantly increased cervical risk among Asians, but not Caucasians. These data suggested that the *IL-6* -174G>C polymorphism may have different effect in different ethnicities. To note, we failed to detect significant relationship among subgroup of HWE > 0.05, but HWE < 0.05. The insufficient statistical power caused by relatively small number of studies should be considered.

Limitations of the current study were listed as follows. First, our pooled results may be biased by unmeasured or residual confounders in the original studies. Thus it also should be cautious about the role of *IL-6* -174G>C in cervical cancer, because age, smoking and drinking status, exposing factors, and gene–environment interactions were not fully adjusted in original studies. Second, some of the subgroup analysis only contained two studies; therefore, the subgroup analyses were not fully implemented for the validity of conclusion was impaired. Third, significant between-study heterogeneity was detected in some comparisons, which might impair the strength of the conclusions. Fourth, the racial bias could not be eliminated as most of the studies included were performed in Asians and Caucasians in this research. The conclusion should be tested before applied to other populations again, due to the genetic and geographical differences.

## Conclusion

In conclusion, our results indicate that the C genotype of *IL-6* -174G>C polymorphism might be associated with higher cervical cancer risk. Our conclusion further helps to explain the etiology of cervical cancer. Yet, further case–control investigations with standardized unbiased design and larger sample sizes are warranted to confirm our findings.

## Abbreviations

CI: confidence interval
HWE: Hardy–Weinberg equilibrium
IL-6: interleukin-6
OR: odds ratio
